# A systematic review of the effectiveness of non- health facility based care delivery of antiretroviral therapy for people living with HIV in sub-Saharan Africa measured by viral suppression, mortality and retention on ART

**DOI:** 10.1186/s12889-021-11053-8

**Published:** 2021-06-10

**Authors:** Mohammed Limbada, Geiske Zijlstra, David Macleod, Helen Ayles, Sarah Fidler

**Affiliations:** 1 Zambart House, PO Box 50697, UNZA-Ridgeway Campus, Lusaka, Zambia; 2 Imperial College London, London, UK; 3 MRC Tropical Epidemiology Group, London School of Hygiene and Tropical Medicine, London, UK; 4 Imperial College and Imperial college NIHR BRC, London, UK

**Keywords:** Human immunodeficiency virus, Antiretroviral therapy, Sub-Saharan Africa, Community-based delivery

## Abstract

**Background:**

Alternative models for sustainable antiretroviral treatment (ART) delivery are necessary to meet the increasing demand to maintain population-wide ART for all people living with HIV (PLHIV) in sub-Saharan Africa. We undertook a review of published literature comparing health facility-based care (HFBC) with non-health facility based care (nHFBC) models of ART delivery in terms of health outcomes; viral suppression, loss to follow-up, retention and mortality.

**Methods:**

We conducted a systematic search of Medline, Embase and Global Health databases from 2010 onwards. UNAIDS reports, WHO guidelines and abstracts from conferences were reviewed. All studies measuring at least one of the following outcomes, viral load suppression, loss-to-follow-up (LTFU) and mortality were included. Data were extracted, and a descriptive analysis was performed. Risk of bias assessment was done for all studies. Pooled estimates of the risk difference (for viral suppression) and hazard ratio (for mortality) were made using random-effects meta-analysis.

**Results:**

Of 3082 non-duplicate records, 193 were eligible for full text screening of which 21 published papers met the criteria for inclusion. The pooled risk difference of viral load suppression amongst 4 RCTs showed no evidence of a difference in viral suppression (VS) between nHFBC and HFBC with an overall estimated risk difference of 1% [95% CI -1, 4%]. The pooled hazard ratio of mortality amongst 2 RCTs and 4 observational cohort studies showed no evidence of a difference in mortality between nHFBC and HFBC with an overall estimated hazard ratio of 1.01 [95% CI 0.88, 1.16]. Fifteen studies contained data on LTFU and 13 studies on retention. Although no formal quantitative analysis was performed on these outcomes due to the very different definitions between papers, it was observed that the outcomes appeared similar between HFBC and nHFBC.

**Conclusions:**

Review of current literature demonstrates comparable outcomes for nHFBC compared to HFBC ART delivery programmes in terms of viral suppression, retention and mortality.

**PROSPERO number:**

CRD42018088194.

**Supplementary Information:**

The online version contains supplementary material available at 10.1186/s12889-021-11053-8.

## Background

There are an estimated 37.9 million people living with human immunodeficiency virus (HIV) globally and 32 million people have died from AIDS-related illnesses since the start of the epidemic [1]. The HIV epidemic has disproportionately affected Africa, particularly sub-Saharan Africa (SSA) which has the largest burden of the disease. Although the region accounts for approximately 6.2% of the world’s total population, it is home to over 50% (20.6 million) of the total number of PLHIV globally, with over 800,000 new infections recorded in 2018 [2].

Antiretroviral therapy (ART) controls viral replication to below the limit of detection and in doing so, improves survival [3, 4] and limits the risk of onward viral transmission [5, 6], but requires daily lifelong adherence to oral medication. Stopping ART invariably leads to rapid viral recrudescence and reversal of its beneficial effects [7]. In order to significantly reduce the number of new HIV infections globally, UNAIDS in 2014 set coverage targets by 2020 for the three key indicators; knowledge of HIV status for 90% of people living with HIV (PLHIV), ART access for at least 90% of all PLHIV and viral suppression for at least 90% of all of those living with HIV on ART; the “90–90-90 targets” with the aspiration to end the HIV epidemic by 2030 [8]. Following the World Health Organization (WHO) 2015 recommendation of lifelong ART for all PLHIV regardless of CD4 count and clinical staging [9], there has been substantial progress in scaling up ART programs; and by mid-2018, 84% of low- and middle-income countries had adopted these guidelines [10, 11] to provide universal treatment to all PLHIV. Despite the high HIV burden, SSA has made tremendous progress in treatment coverage and by 2018, 85% of PLHIV were aware of their status and 67% (13.8 million) were on treatment [12, 13].

Maintaining this unprecedented scale-up of ART services poses a challenge in high HIV burden resource limited settings, especially in SSA where healthcare facilities are overburdened with long waiting times, inadequate and overburdened human resources, transportation costs, congestion and long waiting times at the health facility-based care (HFBC) [14, 15], leading to poor retention in care and adherence. Recent data from sub-Saharan Africa (SSA) shows 5- year retention on ART is close to 60% [16–21].

Decentralizing ART provision services outside of the HFBC into the communities holds the promise of improving the continuum of care and facilitating access to treatment. Various models of non-health facility-based care (nHFBC) [22] have been piloted and implemented in high burden low resource settings and are now being increasingly recognised as safe and effective alternatives to the current standard model of health facility-based care in SSA [23, 24]. These include; healthcare worker-managed groups (adherence clubs); client managed group models (community adherence groups (CAGs)); and out-of-facility individual models (community-based distribution points (CBDPs) and home-based delivery). Adherence clubs consists of a group of 15–30 stable PLHIV who meet up at a venue within or outside the HFBC space, once every 2–3 months where they receive their adherence support and pre-packed medications by a trained lay worker or healthcare worker. Club members are seen once or twice-yearly at the clinic for routine clinical review and laboratory tests [25–29]. CAGs, originally developed by Médecins Sans Frontiéres (MSF) in Tete, Mozambique, also target stable patients who receive ART refills and adherence support in a group, where each member of the group takes turns collecting ART for all group members. Each group is composed of approximately six patients who meet up every 2–3 months, and each member has their routine clinical visit once or twice-yearly [26, 30–32]. Out-of-facility models vary according to the services delivered, by whom and where in the community these services are provided. In home-based delivery, clients receive their adherence support and pre-packed medications once every 3 m in their homes by a trained lay worker [33, 34]. CBDPs allow patients to pick up their drug refills at a designated place in the community [26, 27, 35, 36].

These models of care are best directed towards stable adult patients, defined as those with suppressed HIV viral loads on ART for more than 6 m. It allows them to receive treatment and sometimes medical care within their communities with ongoing adherence support where needed, and may sometimes involve community health workers (CHWs) dispensing pre-packedART, thus reducing the frequency of clinic visits.

Ideal nHFBC models of ART delivery must be sustainable and safe. They must confer similar successful clinical outcomes in order to effectively contribute to the decrease of HIV transmission and extension of life expectancy. Feasibility of these models need to be stringently evaluated and compared with concurrent HFBC in order to determine the safe sustainable delivery of ART to UNAIDS targets. Several systematic reviews published recently have shown that community programs increase both affordability and accessibility to ART [24] and have shown that there are no significant differences in optimal ART adherence, virological suppression (VS), all-cause mortality and loss-to follow-up (LTFU) between patients assigned to nHFBC and HFBC [23, 37]. This review looks at programmatic data and trials from 2010 onwards in order to provide an update on large amounts of recently published data, as several models have been rolled out providing more data on clinical outcomes.

We undertook a review of published literature comparing HFBC with nHFBC models of ART delivery in terms of health outcomes; viral suppression, loss to follow-up, retention and mortality among PLHIV. We included all descriptions of novel programmatic delivery of ART in nHFBC settings, and compared where available specific outcomes between HFBC and nHFBC, including VS, mortality, retention and LTFU.

## Methods

### Search strategy

A systematic electronic search of peer-reviewed literature was conducted most recently on the 21 August 2019 in the following databases: Medline, Embase and Global Health. The search strategy was created with the support of a medical librarian; key terms were identified to combine ART AND nHFBC AND SSA. The search strategy is outlined in full in Additional file 1: Appendix 1. The review was prospectively registered with online database PROSPERO (ID=CRD42018088194). In addition to the databases, two key UNAIDS reports and all WHO guidelines, and their references, from 2010 onwards were reviewed.

### Eligibility criteria

Articles were considered for inclusion if they described the effectiveness of one of four nHFBC methods of delivery of ART in sub-Saharan African settings: adherence clubs, CAGs, CBDPs and home-based delivery. Adherence clubs were included irrespective of whether they were physically located within the healthcare facility or in the community as they are run independently and are considered novel care pathways outside the routine HFBC pathway. Appointment spacing, and fast track refills that take place within the facility were excluded as this was considered to be part of standard HFBC pathway. Studies had to measure a clinical outcome, either; retention in care, LTFU in accordance with WHO and national guidelines definitions, transfer to alternative care, viral load (VL), viral suppression (VS), CD4 count or mortality. The definition of LTFU varied by study and year, but papers were considered eligible if they defined LTFU in accordance with standard WHO and guideline practices [38]. While some studies reported patient outcomes within the LTFU cohort, such as death or transfer to other services, this was not essential for inclusion. The definitions of viral suppression were varied between studies as laboratory assays changed, but for this analysis we included all papers that reported to < 1000 copies HIV RNA/mL.

For inclusion, studies were not required to have a comparator current standard of care control group. It was not necessary for studies to be delivering ART in isolation of other interventions, such as counselling. There was no restriction on study population age, history of infection or line of ART.

Original research articles were included, and systematic reviews were excluded. Where data from the same cohort was published multiple times, the most recently available publication was included. The search was conducted in English only due to available expertise, time and budgetary restrictions. A publication date limit of 1st January 2010 until 31st August 2019 was applied to the searches in all databases as the aim was to review the current published literature and update previously published review articles [24].

### Data extraction and quality appraisal

**Fig. 1:**
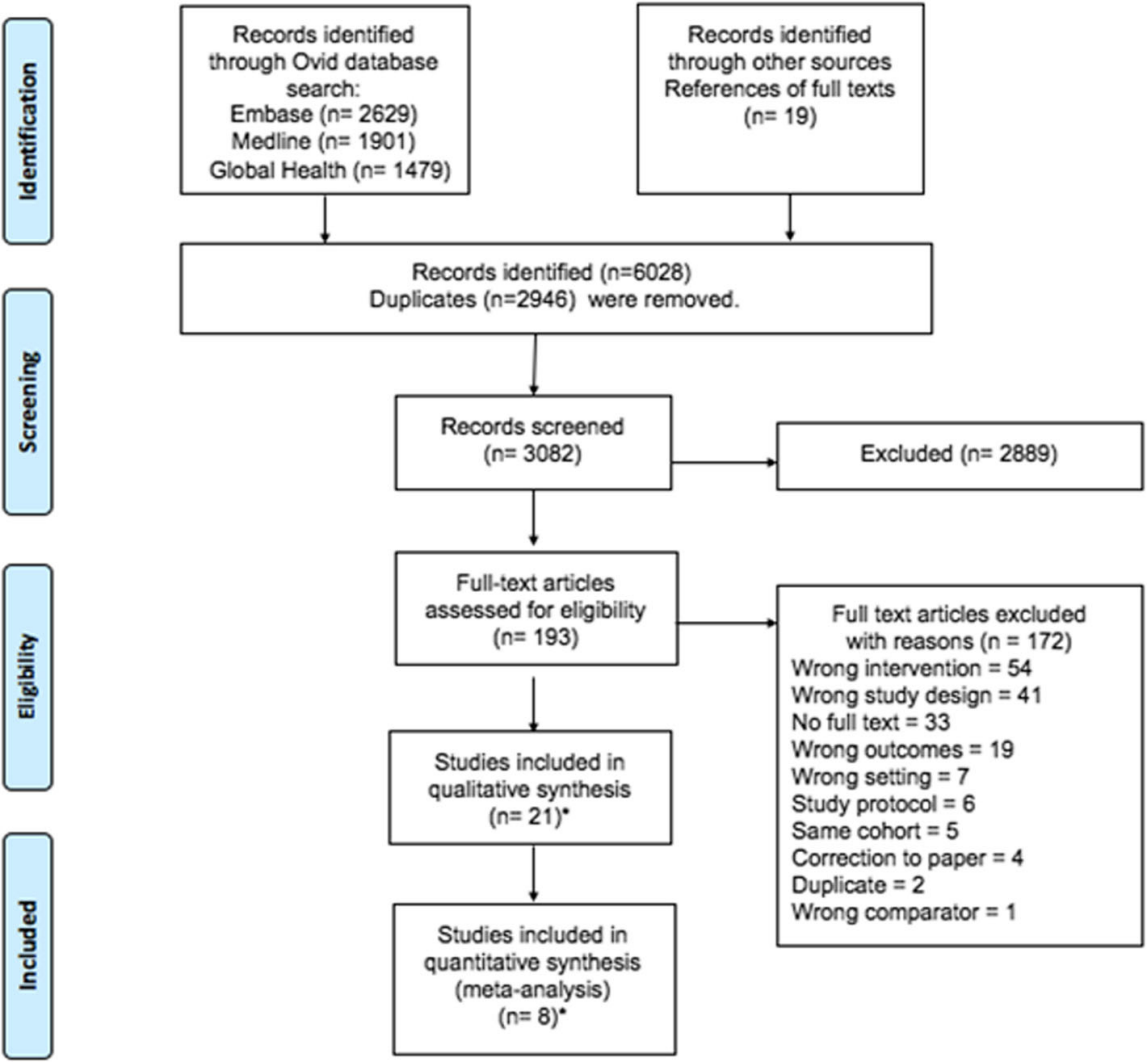
PRISMA flowchart of search strategy

All database search results were imported into EndNote software (EndNote X8.2) for duplicate removal, and then into Covidence systematic review software, which was used for screening [39]. The screening of titles and abstracts and the full text reviews of eligible articles were done in duplicate by two independent reviewers (GZ, ML). All conflicts were resolved through discussion between both reviewers, and a third reviewer (SF). Where full texts of abstracts were not available, these were accessed via the British Library. Additional articles were identified by examining references of articles included for full text review (Fig. 1). Articles considered eligible for inclusion were read in full by GZ & ML, and approved by reviewer SF.

Data was extracted in duplicate by two reviewers (GZ, ML), including: first author, year of publication, country of origin, study design, sample size, the community model used to deliver ART, outcomes, length of follow up and who was responsible for ART provision. All discrepancies in data extracted were solved through discussion between both reviewers. Results of this data extraction were summarized in Table 1. Quality analysis was done by reviewers GZ and ML using the Cochrane tool for risk of bias for all randomised control trials (RCTs) and using the Newcastle-Ottawa scale [58, 59] for cohort studies, which can be found summarised in Appendices 2 and 3.

**Table 1:** Characteristics of the studies and their Design, nHFBC model and key findings

Study	Setting	Non-facility based model	Comparator	Sample Size	Length of follow-up	Outcomes and key findings
**RANDOMIZED CONTROL TRIALS**						
Fox 2019 [37]	South Africa	Adherence clubs	Health care facility	*N* = 596 AC *n* = 275 HCF *n* = 294	18 months	***Viral Suppression*** – comparable 12 months viral suppression between the intervention (80%) and control (79.6%) arms (aRD: 3.8%; 95% CI: −6.9 to14.4%). ***Retention*** – AC’s had a higher 1-year retention (89.5% vs 81.6%, aRD:8.3%; 95% CI: 1.1 to 15.6%)
Hanrahan 2019 [40]	South Africa	Community Adherence clubs	Health care facility clubs (Standard of care)	*N* = 775	24 months	***Loss from the club*** – proportion of patients who dropped out of clubs in both community and facility clubs or were transitioned to standard of care. Overall, 47% [95%CI 44–51%] of patients were returned to health care facility. Among community-based club participants, the cumulative proportion lost from club-based care was 52% (95% CI: 47–57%), compared to 43% (95% CI: 38–48%, *p =* 0.002) among clinic-based club participants. ***Virological failure -*** Documented viral rebound was higher among participants assigned to facility-based clubs (21, 95% CI 13–27%) than those assigned to community-clubs (13, 95% CI 8–18%, *p* = 0.051). But this was not significant. ***All-cause mortality –*** no mortality observed in both arms **Loss from ART care** -during follow up, 77 (10%) overall. No significance between the two arms. Among community club participants, the proportion lost from any ART care was 12% (95% CI 9–16%), compared to 7% (95% CI 5–10%, *p =* 0.024) among facility- club participants, corresponding to a difference of 5% (95% CI 1–9%, *p =* 0.018). In a univariate Cox proportional hazards model, the risk of loss to any ART care was non-significantly increased among participants assigned to community clubs as compared with those assigned to facility clubs (HR 1.69, 95% CI 0.98–2.91, *p =* 0.057).
Geldsetzer 2018 [41]	Tanzania	Home ART delivery	Health care facility	*N* = 2172 HD *n* = 1163 HCF *n* = 1009	326 days	***Virological failure*** – 10.9% (95/872) in the control arm and 9.7% (91/943) in the intervention arm were failing at the end of the study period. Risk ratio demonstrated non-inferiority of the HBC to HCF (RR 0.89 [1-sided 95% CI 0.00–1.18]) ***Lost to follow-up*** – 18.9% in HBD versus 13.6% in HCF. No *P* value or CI reported. **Mortality** – 0.09% in HBD versus 0.2% in HCF. No P value or CI reported.
Woodd 2014 [42]	Uganda	Home ART delivery	Health care facility	*N* = 1453 HD n = 859 HCF *n* = 594	28 months	Home delivery of ART and support leads to similar survival rates as clinic-based care. ***Mortality –*** One hundred and ninety-seven participants died over a median follow-up time of 28 months (IQR 15–35) giving an overall mortality rate of 6.36 deaths per 100 person-years [95% confidence interval (CI) 5.53–7.32]. 110 (25%) deaths in participants with baseline CD4 < 50 cells and 87 (9%) in those with higher baseline CD4.Among participants with baseline CD4^+^ count < 50 cells/μl, mortality rates were similar for the home and facility-based arms; adjusted mortality rate ratio 0.80 [95% confidence interval (CI) 0.53–1.18] compared with 1.22 (95% CI 0.78–1.89) for those who presented with higher CD4^+^ cell count. In CD4 counts < 50 cells – crude mortality RR 0.81 and In CD4 counts higher - crude mortality RR 0.55 ***Lost to follow up*** – 1.8% among those with CD4 < 50 and 2.6% among those with CD4 at least 50.
Amuron 2011 [43]	Uganda	Home deliveries	Health care facility	HD n = 594 HCF *n* = 859	42 months	***Mortality*** – in the facility there were 117 deaths (mortality rate 6.3 per 100 persons per yrs.) whereas in HBD, 80 deaths (mortality rate 6.5 per 100 person yrs.). The one, two and three year survival probabilities (95% CI) were 0.89 (0.87–0.91), 0.86 (0.84–0.88) and 0.85 (0.83–0.87) respectively
Selke 2010 [44]	Kenya	Home ART delivery	Health care facility	HD *n* = 96 HCF *n* = 112	28 months	Home delivery of ART and support resulted in similar clinical outcomes as clinic care but with half the number of clinic visits. Task-shifting and mobile technologies can deliver safe and effective community-based care to PLHIV. ***LTFU*** – 4.5% in the HCF and 5.2% in Home delivery [95% CI: 0.24 to 3.03; *p* = 1.0] ***Mortality*** – 0 in both arms ***Viral rebound –*** no significant difference between the two groups (10.5% in HBD and 13.5% in HCF, 95%CI: 0.54 to 3.31, *p* = 0.65)
**OBSERVATIONAL COHORT STUDIES**						
Fox 2019 [37]	South Africa	Decentralized medication delivery (DMD)	Health care facility	*N* = 578 DMD *n* = 232 HCF *n* = 346	18 months	
Tun 2019 [45]	Tanzania	Community Based ART distribution (CBPDs)	Health care facility	CBPD *n* = 309 HCF *n* = 308	6 months	**Retention** in the CBDP – 82.8% vs 82.1% in the HCF at 6 months **LTFU** – 53 in the intervention and 55 in the HCF arms
Pasipamire 2018 [46]	Swaziland	1. Community Adherence groups (CAGs) 2. Facility Based clubs 3. Treatment outreach	No comparator	***N*** **= 918** CAGs *n* = 531 FBC *n* = 289 Outreach *n* = 98	12 months	**Retention in the models** – The overall care model retention was 90.9 and 82.2% at 6 and 12 months. Retention in the care models differed significantly by model type, being lowest in CAGs at all time points (*p* < 0.001). Only 70.4% of patients were retained in CAGs at 12 months compared with 86.3% in comprehensive outreach and 90.4% in clubs. Retention in care model was significantly higher in eligible patients compared with non-eligible patients (85.0 and 76.4% at 12 months, *p* = 0.017. **Retention to ART –** over 90% from all three models and no difference noted (*p* = 0.52).Patients in CAGs had a higher risk of disengaging from the care model (aHR 3.15, 95%CI: 2.01–4.95, *P* < 0.001) compared with treatment clubs. Note: disengagement defined as LTFU, Death, return to clinical care)
Myer 2017 [47]	South Africa	Adherence clubs [post-partum women]	Health care facility	*N* = 110 AC n = 77 HCF *n* = 33	6 months post-partum follow-up	**Viral suppression -** overall no difference in viral suppression between the two groups. **86% of women remained in the evaluation through 6 months postpartum; in this group, there were no differences in VL < 1000 copies/mL at six months postpartum between women choosing HCFs (88%) vs. adherence clubs (92%;** ***p*** **= 0.483.**
Vogt 2017 [48]	Democratic Republic of Congo (DRC)	Community based refill centers	No comparator	*N* = 2259	24 months	Attrition increased steadily after decentralizing services such as drug pick up points. Low attrition throughout follow-up ***LTFU*** – 9.0% at 24 months ***Mortality –*** 0.3% at 24 months overall attrition was 5.66/100 person years (95% CI: 4.97 to 6.45)
Tsondai 2017 [49]	South Africa	Adherence clubs	No comparator	*N* = 3216	24 months	Stable patients on ART can safely be offered differentiated care as they overall had good outcomes. Adherence clubs scaled up at large scale had had high levels of retention and viral suppression. ***Retention*** – Retention was 95.2% (95% CI: 94.0–96.4) at 12 months and 89.3% (95% CI: 87.1–91.4) at 24 months after AC enrolment. ***Viral suppression -*** Of the 88.1% who had a viral load assessment, 97.2% (95%CI, 96.5–97.8) were virally suppressed < 400 copies/ml ***LTFU*** – 4.2% (135). Cumulative incidence of LTFU was 2.6% (95% CI, 2.1–3.2) at 12 months, rising to 6.9% (95%CI, 5.7 to 8.1) at 24 months after AC enrolment. ***Mortality*** – 0.1% (95% CI, − 0.01 to 0.2) at 12 months and 0.2% (95%CI, − 0.01 to 0.4)
Decroo 2017 [50]	Mozambique	Community ART groups (CAGs)	Health care facility	CAGs *n* = 901 HCF *n* = 1505	24 months	***LTFU*** – overall 12% [11.2% in HCF and 0.8% in CAGs]. CAG members had a greater than fivefold reduction in risk of dying or being LTFU (adjusted HR: 0.18, 95% CI 0.11 to 0.29). **Retention -** 12-month and 24-month retention in care from the time of eligibility were 89.5 and 82.3% respectively among patients in individual care and 99.1 and 97.5% among those in CAGs (*p* < 0.0001).
Auld 2016 [51]	Mozambique	Community support ART groups (CASG)	Health care facility	*N* = 306,335 CASG *n* = 6766 HCF *n* = 299,569	4 years	***Mortality*** – similar rates in both groups [0.3% among CASG at 2 yrs. and 1.4% at 4 yrs.] CASG patients were associated with a 35% lower LTFU rates [AHR 0.65; 95% CI:0.46, 0.91] but similar mortality.
Grimsrud 2016 [52]	South Africa	Adherence clubs	Health care facility	*N* = 8150 AC *n* = 2113 HCF *n* = 6037	12 months	**Viral suppression** – high rates of VLS among those who had a VL result, but no comparison made between the two cohorts. ***LTFU –*** clubs were associated with a decreases risk of LTFU compared to facility in all crude and adjusted models. Clubs were associated with a 67% reduction in LTFU compared with facility (aHR 0.33, [95% CI, 0.27–0.40]).
Okoboi 2016 [53]	Uganda	Community based distribution points (CBDP)	Health care facility	CDDP *n* = 476 HCF *n* = 752	5 years	Overall retention rates were above 80% in both HCF and CBDP ***Retention rates*** – 83.9% in the facility and 82.9% retained in the community distribution model of delivery (*p* = 0.670)
Jobarteh 2016 [54]	Mozambique	Community ART support groups (CASG)	Health care facility (non-CASG)	CAGs *n* = 6760 HCF *n* = 123,178	12 months	**LTFU** – LTFU among CASG and non-CASG members was 7.2 and 15.9%, respectively. Compared with CASG participants, non-CASG participants had significantly higher LTFU (hazard ratio [HR]: 2.36; 95% confidence interval [CI]: 1.54–3.17; *p* = .04] ***Mortality -***no significant mortality differences between CASG and non-CASG members (1.4% vs 1.2%) (HR:0.98; 95%CI, 0.14 to 1.82; *p* = 0.96)
Okoboi 2015 [36]	Uganda	Community distribution points (CDDP)	No comparator	CDDP *n* = 3340	5 years	Community-based ART distribution systems are capable of overcoming barriers to ART retention and result in good rates of virologic suppression. ***Viral suppression-*** of the 870 patients who had a VL measured, 87% were suppressed **Mortality-** mortality rate was low (3.22 per 100 person-years) ***LTFU-*** 1.59 per 100 person-years ***Retention***- more than 69% of patients who initiated ART from 2004 to 2009 were retained in care after more than 5 years of treatment.
Decroo 2014 [32]	Mozambique	Community ART groups (CAGs)	No comparator	CAGs *n* = 6158	4 years	Long-term retention in CAG was exceptionally high [91.8% at 4 years of follow-up (95% CI, 90.1 to 93.2)]. ***LTFU*** – event rate was 0.1% per 100-person yrs. ***Mortality*** – event rate was 2.1 per 100-person yrs. ***Retention*** among CAG members at 1 year on ARTwas 97.7% (95% CI 97.4–98.2); at 2 years, 96.0% (95% CI 95.3–96.6); at 3 years, 93.4% (95% CI 92.3–94.3); and at 4 years, 91.8% (95% CI 90.1–93.2). Overall, the attrition rate was 2.2 per 100 person-years among the 5729 adult members.
**Study**	**Setting**	**Non-facility based model**	**Comparator**	**Sample size**	**Length of follow-up**	**Key outcomes**
Luque-Fernandez 2013 [55]	South Africa	Community Adherence clubs	Health care facility	ACs *n* = 502 HCF *n* = 2372	3 years	Outcomes less frequent in patients participating in the clubs. ***Viral rebound*** – 214 patients had viral failure at study end in the HCF (90.4 event rates per 1000 person yrs. [95%CI: 79.1–103.4). In the clubs 14 had viral rebound 31.8 event rates per 1000 person yrs. ***Retention*** - 97% of club patients remained in care compared with 85% of other patients. In adjusted analyses club participation reduced loss-to-care by 57% (hazard ratio [HR] 0.43, 95% CI = 0.21–0.91). ***Mortality + LTFU -*** 12.8% of patients were LTF or had died (323 LTF and 40 deaths). Both outcomes were less frequent for patients participating in the clubs (29.8 vs 116.8 per 1000 person-yrs. for LTFU/death, crude rate ratio [RR = 0.25, 95% CI 0.14–0.41]
Kipp 2012 [56]	Uganda	Home based ART delivery	Health care facility	HBD *n* = 185 HCF *n* = 200	24 months	ART outcomes such as viral suppression in community models were equivalent to those receiving care in the facility. ***Viral suppression –*** patients in the home delivery model were 2.47 times more likely to achieve viral suppression compared to those in the facility based [95% CI for OR 1.02–6.04 *p* = 0.046]. ***Mortality*** – 32(17%) in Home delivery vs 23 (12%) in HCF. This had limitations as the LTFU in both groups includes unknown number of deaths. Crude mortality was higher in the HBD cohort compared to the HCF cohort, though this difference was not statistically significant (17.3% vs. 11.5%, *p* = 0.10). ***Retention −*** 70% in home model vs 71% in facility
**CROSS-SECTIONAL STUDY**						
Chimukangarta 2017 [57]	Zimbabwe	Outreach ART delivery	No comparator	*N* = 143	18 months	***Viral suppression-*** over the course of the study period, 94% were virally suppressed

### Quantitative and qualitative analysis

Results were extracted for VS (thresholds defined in the articles ranged from ≤1000–400 copies HIV RNA/mL), mortality and LTFU/retention in care. Studies with variable definitions of VS were still considered eligible for quantitative comparison. Pooled estimates of the comparison between nHFBC and HFBC were calculated for both VS and mortality using random-effects meta-analysis. When comparing VS, the pooled risk difference was the reported statistic, and for mortality the pooled hazard ratio was reported. Due to the large variations in the definitions of LTFU and retention in care between papers, only a descriptive analysis was carried out in accordance with the Synthesis without meta-analysis (SWiM) in systematic reviews: reporting guideline [60]. For quality assessment, RCTs were risk assessed using the Cochrane Risk of Bias tool [59] which can be found in full in Additional file 2: Appendix 2. Quality assessment of cohort studies was done using the Newcastle-Ottawa Scale (Additional file 3: Appendix 3) [58].

## Results

Our search identified 3082 non-duplicate records, of which 2889 were excluded after abstract and title screening against our search criteria. One hundred ninety-three records were eligible for full text screening, of which 21 published papers were eligible for inclusion in our analysis (Fig. 1).

Of the 21 articles included, results were presented from a total of six randomized control trials (RCTs) [37, 40–44], 15 observational cohort studies [32, 36, 37, 45–56] and one cross-sectional study [57] (one article presented the results from both an RCT and a cohort study). These studies were conducted in SSA, including: South Africa, Uganda, Tanzania, Mozambique, Kenya, Zimbabwe, Eswatini and Democratic republic of Congo. The number of participants included in the studies ranged from 129 to 129,936, and the design and methodology of the included studies are detailed in Table 1. Our included articles represented nHFBC models that provided service delivery either as individual or group models outside the healthcare facility including facility or adherence clubs, home-based delivery, community adherence groups or distribution points and outreach ART delivery (Table 1). ART delivery was done by a range of community healthcare workers, volunteers and nurses.

The six randomised control trials were appraised using the Cochrane tool for risk of bias. Sequence generation and allocation concealment were well conducted, and risk of bias was low amongst the studies. Blinding of participants and personnel was not possible in any of the studies due to the nature of the intervention, but there was variability amongst blinding of outcome assessors as in some cases the assessors were also involved in project management. The data collected however were generally objective measures obtained from medical records, which is at minimal risk of bias, even for assessors who were informed of patient allocation. Not all RCTs had published study protocols, which increases the risk of selective outcome reporting, but all did report numbers of attrition and mortality, minimising risk from incomplete outcome data.

### Virological suppression (VS) and viral load (VL)

**Fig. 2:**
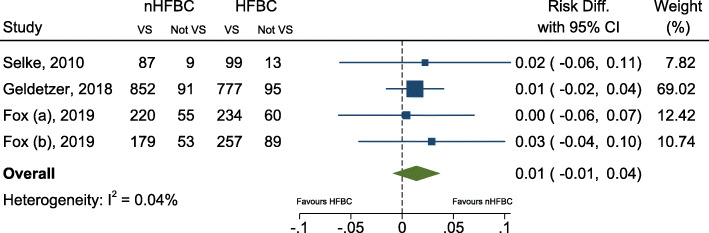
Forest plot for estimated pooled risk difference comparing viral suppression among those receiving health facility-based care (HFBC) and non-health facility-based care (nHFBC), including results only from randomized controlled trials. Legend to the figure: Dashed line represents zero risk difference. VS (virally suppressed)

From our included studies, 10 out of 21 reported VS or HIV viral load rebound as an outcome measure. Of these, three articles [36, 49, 57] did not compare to a facility-based cohort and were therefore excluded from the pooled analysis. Three articles [37, 41, 44] were RCTs that compared outcomes to a facility-based cohort, one of which [37] included results from two separate RCTs published in the same article. The remaining four studies were all observational cohort studies [47, 52, 55, 56] comparing VS among participants receiving community-based care with those receiving facility-based care. The pooled risk difference of virological suppression amongst RCTs are shown in Fig. 2, and including the observational studies are shown in Additional file 4: Appendix 4. There was a remarkably consistent effect (I^2^ = 0.04%) found across the four randomized trials, very marginally in favour of community care, with an overall estimated risk difference of 1% [95%CI -1, 4%). There was no statistically significant evidence (*p* = 0.24) of a difference in viral suppression between the two groups. The definition of viral suppression varied between studies, with Geldsetzer et al. using < 1000 copies/ml, Fox at al using < 400 copies/ml, and Selke not defining it. The viral load or suppression reported at baseline in these RCTs also varied. Geldsetzer reported the percentage of people with VL < 1000 copies/ml or CD4 < 350 cells/μl (which was 17.4% in control and 15.4% in intervention group),**.** Fox reported the median viral load (copies/ml) and interquartile range. For the adherence club (AC) control and intervention groups, these were 50 (20–124) for both and in the Decentralized Medication Delivery (DMD) control and intervention groups these were 42 (20–100) and 124 (35–124) respectively. Selke reported the proportion with detectable viral load at baseline, which was 8.5% in the intervention and 12.6 in the control group. These were all studies assessed as high quality, and apart from not being blinded, all had an overall low risk of bias. Three of the four observational studies showed results broadly consistent with the randomised trial results (although slightly more favourable towards nHFBC, with risk differences ranging from 4 to 6%). One study by Grimsrud et al. had results showing much greater viral suppression in nHFBC (estimated risk difference of 39%), although in that paper the patients receiving nHFBC were those who were classed as “stable on ART” and the comparison group were not (adjusted results for VS were not presented in the paper) [52].

### Mortality

**Fig. 3:**
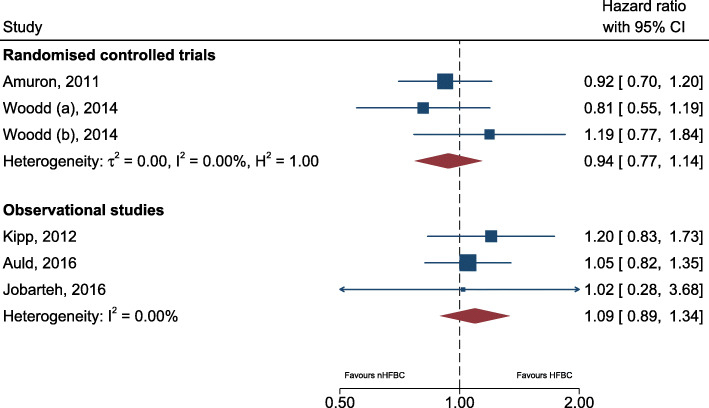
Forest plot for estimated pooled hazard ratio comparing mortality among those receiving health facility-based care (HFBC) and non-health facility based care (nHFBC). Legend to the figure: dashed line represents hazard ratio of 1. *Wood (**A**) is among participants with a baseline CD4 count < 50, while Woodd (**B**) is among participants with a CD4 count of 50 +

Nine papers were identified that reported mortality, four of which were RCTs. Only two RCTs [42, 43] did a formal comparison between trial arms on mortality. The two RCTs included in the pooled analysis were rated as fair quality, and reported the results stratified by whether baseline CD4 count was less than or greater than 50, increasing the accuracy of the intervention comparison. The other two RCTs reporting extremely low rates of mortality (Selke et al. reported no deaths in HFBC and one in nHFBC and Geldsetzer et al. reported two in HFBC and one in nHFBC). Of the five observational cohort studies, four reported a formal comparison of mortality. The hazard ratios across all studies ranged from 0.8 up to 1.2, but with all confidence intervals crossing the null of HR = 1 (Fig. 3). This resulted in a pooled estimate equal to 1.01 (95% CI 0.88–1.16), providing no evidence (*p* = 0.92) of a difference in the mortality rate among those not in facility-based care compared to those in facility-based care.

Due to the large amount of heterogeneity, results were described for LTFU and retention in care without formal methods of statistical comparison. Instead, data was tabulated comparing reported outcomes. A page referencing guide for the SWiM guideline for these outcomes can be found in Additional file 5: Appendix 5.

### Loss to follow-up

A total of 15 studies reported LTFU as an outcome, of which four were from RCTs and 11 from observational cohort studies and are summarized in Table 1 and Additional file 6: Appendix 6. In most studies LTFU was defined as no longer having contact with the care services, but there was a large degree of variability in the time frame. This commonly ranged from 60 days to 6 months, however multiple studies defined LTFU as no visit or contact with the service during the study period, which was up to 5 y. Additionally, there were varying degrees of investigation into outcomes of the LTFU populations, with some studies documenting mortality and transfer to alternative services, and some not documenting any.

In the studies included, there were four RCTs that included LTFU as an outcome where LTFU was defined as outlined in Additional file 6: Appendix 6 and varied between studies [34, 40, 42, 44]. A cluster RCT undertaken in South Africa comparing adherence clubs with healthcare facility clubs over a 24 months period showed 105 patients were LTFU with no significant difference between the two study arms [40]. Their definition varied from those used in other studies as it was a measure of loss from their intervention, which goes beyond missing visits, but also includes patients who developed any of the exclusion criteria, such as comorbidity or viral rebound. A RCT in Tanzania [41] compared home delivery to HFBC, and defined LTFU as not having a VL measurement after enrolment into the model of care over the entirety of the 12-month study period. They demonstrated non-inferiority in the rates of LTFU. In the other two randomised trials by Selke et al. and Woodd et al., LTFU was defined as not having had contact with the care services during the study period, which was 28 months in both studies. Selke et al. [44] compared home delivery model to healthcare facility in Kenya and showed comparable LTFU outcomes (4.5% in HFBC versus 5.2% in home delivery) and similarly, Wood et al. compared home delivery to HFBC in Uganda and demonstrated similar rates of LTFU, which were 2.36% in the facility and 2.33% in the community [42].

Eleven observational studies reported LTFU with varying definitions. Six of these studies did a comparison between nHFBC and HFBC and showed nHFBC had comparable or better LTFU outcomes compared to HFBC [45, 50–52, 54, 55] (Additional file 6: Appendix 6: Table 4). Among these, LTFU was defined as being late for their scheduled pharmacy pick-up date by either 60, 90 or 180 days late with the exception of Tun et al. who defined LTFU as combined mortality, transfer out and withdrawal [45]. Grimsrud et al. showed community adherence clubs were associated with a reduction in the risk of LTFU compared with the clinic with a two-third reduction in the hazard of LTFU [52] (Additional file 6: Appendix 6). Luque-Fernandez et al. compared adherence clubs to healthcare facility and demonstrated that a combined outcome of time to either death or LTFU was less frequent in club participation than in the facility (crude RR 0.25 95%CI: 0.14, 0.41) [55]. Similarly, in Mozambique, patients who participated in community adherence support groups were associated with a lower LTFU rates as compared to those who did not participate in these groups. Auld et al. showed participating in CAGs was associated with a 35% lower LTFU rates (AHR 0.65; 95%CI: 0.46, 0.91) [51]. Another study comparing CAG to non-CAG showed higher LTFU rates amongst non-CAG members (HR 2.36 95%ci: 1.54, 3.17) [54]. In the same country, a comparison between CAGs and HFBC showed that CAG members had a greater than 5-fold reduction in the risk of combined LTFU and mortality (adjusted HR 0.18 95%CI: 0.11, 0.29) [50]. A total of five studies had no comparison to HFBC [32, 36, 46, 48, 49] and despite varying definitions of LTFU, a study in South Africa showed a cumulative incidence of LTFU at 2.6 and 12.2% at 12 and 24 months respectively [48, 49] (Additional file 6: Appendix 6: Table 5).

The definition of LTFU varied amongst included studies, including a missed scheduled visit, being late for drug pick-ups or withdrawal from a model, which could include death or patients transition to alternative health care facility. For studies that defined LTFU as having missed a scheduled visit or model withdrawal, only three indicated patients transition to HFBC [40, 41, 45].

### Retention

A total of 13 studies in our review, two of which were published in the same paper [37], reported retention as an outcome, nine of which provided a comparison to health facility based care (Additional file 6: Appendix 6 Table 4). Three RCTs compared retention between nHFBC and HFBC [37, 41, 44], which showed that the community models had comparable rates to those in the facility. Fox et al. defined retention as those not LTFU, died or transferred to alternative care, and reported 81.6% participants retained in facility and 89.5% participants retained in the community with a risk difference of 7.8% [37]. Selke et al. defined it as those still in care at the end of the follow up period, reporting rates of 91.1% in facility compared to 90.6% in the community [44]. Similarly, Geldsetzer et al. defined attrition as those no longer in care, the inverse rates of which are reported as retention of 86.4% in the facility and 81.1% in the community [41].

Equally, most observational studies demonstrated similar retention outcomes between nHFBC and HFBC [45, 53, 56] or better retention outcomes in nHFBC [50, 54]. Only one study showed better HFBC retention rates [37]. Definitions of retention in care used were similar across all studies, however there was large variation in follow up period, ranging from six months to five years. Among the four studies that did not provide a comparison to HFBC, retention rates for nHFBC generally exceeded 90%, including a study with follow up of four years. A study from 2015 by Okoboi et al. was the exception, reporting a retention rate of 69% in patients on treatment for more than five years [36].

## Discussion

We reviewed articles describing the current evidence of community ART programs taking place in SSA between 2010 and 2019 on the following key outcomes; Viral load suppression, mortality, LTFU and retention. From our review, all the articles that described nHFBC ART programs found evidence that decentralizing HIV services into the community for PLHIV has promising outcomes and is a safe alternative to facility based care programs in resource limited high burden HIV settings for stable PLHIV on ART. Adherence clubs that were physically located within the health-care facility were also considered as nHFBC as they ran independently and thus considered as outside the standard HCF provision. The studies suggest that levels of VS and mortality are similar in both nHFBC and HFBC groups. Similarly, with regards to LTFU and retention, articles included in our review showed comparable or slightly better LTFU and retention outcomes amongst nHFBC models when compared to HFBC. However, whilst we identified 21 articles that described one or more outcomes of nHFBC models in SSA countries, only two-thirds of the articles compared these models to the HFBC, limiting the strength of conclusions that can be drawn.

In all included articles, the primary clinical care provider for these nHFBC models was poorly described, but provision of the core packages such as ART dispensation, adherence support and referrals of sick patients to the clinics was often shared by community or trained lay workers. nHFBC models have shown that decentralizing HIV services into the community may potentially overcome major structural and financial barriers faced by PLHIV to ART initiation and retention [27]. These models are capable of achieving a range of potential additional benefits to healthcare providers and PLHIV on ART, including patient satisfaction, reduced costs, convenient and efficient service delivery and better clinical outcomes and promote healthy behaviors such as decrease alcohol abuse [23]. As the numbers of PLHIV accessing treatment increases following the 2015 WHO ART guidelines [9], nHFBC models have shown the potential to be able to deliver a package of essential ART services beyond the clinic, freeing up the capacity within the HFBC workforce to be able to focus on more complex cases [24].

Our findings suggest that nHFBC programs can achieve favorable outcomes for stable PLHIV on ART in resource limited settings, which is in line with a previously published systematic review by Decroo et al. that looked at community-based intervention programs [24]. This review has updated and summarized the evidence that has been published since Decroo et al’s review in 2013, and proposes that community-based intervention programs can make treatment readily accessible and affordable as well as help support adherence and sustain retention of patients on ART over the long term [24]. In Uganda, Kenya and Tanzania, lay workers or community health workers delivered ART to patients homes [41, 44, 61] whereas in Tete, Mozambique, CAGs were used to deliver ART within the community [50]. Similarly, in South Africa, adherence clubs piloted by MSF equally showed promising results [55].

With respect to other relevant outcomes, studies comparing CD4 count outcomes between HFBC and nHFBC models showed patients in nHFBC models can achieve similar outcomes in terms of CD4 gains [44, 52]. Decroo et al. also included studies analyzing costs of the interventions, and found that provider costs were either similar or lower in nHFBC models, and considerably more cost-effective for patients [24]. Our review did not include cost-analysis as there have been very few studies that have informed on the costs or cost-effectiveness of these nHFBC models. Studies that have reported on costs have found that provider outcomes were similar for HFBC and nHFBC [62, 63]. One study found that community-based intervention programs were much more cost-effective than estimates for facility based care [64]. However, a recent study in Tanzania showed that although patient satisfaction with a home-based program was high and was likely to save patients substantial amount of time, other envisaged benefits of decongesting the healthcare facility and reductions in patients’ health expenditures were minimal [41]. Clearly more research using economic outcomes in different contexts to compare the costs, effectiveness and sustainability of the models are needed. Available data suggests that these models, even if equivalent or significantly non-inferior to the HFBC, may be more cost-effective. Patient transportation costs and use of personnel, operational and utility costs are likely to be lower. This in addition to improved retention rates are more likely to make nHFBC models more cost-effective and sustainable in the long run [23].

At the time of writing, Long et al. published a rapid review of differentiated service delivery models for ART in SSA and noted despite the widespread expectations that these models will be cost-saving, they found little data to support this contention [65]. When evaluating programmatic costs of such nHCFB models of ART delivery, an additional cost that is difficult to measure is the potential costs associated with onward HIV transmission amongst those who interrupt ART with consequent viral rebound.

nHFBC models also have the potential to have an impact on the relationship between healthcare providers and patients and can thus strengthen social and peer support [66]. These models have the opportunity to transform the current siloes to a more integrated approach that will enable HIV care to be combined with care for other conditions, including non-communicable diseases that are becoming more prevalent in resource limited settings [23].

Our study had several limitations and despite searching several databases, yielded a small number of studies that looked at ART delivery for final inclusion. We also noted there is paucity of data from other regions in Africa such as West and Central Africa where the HIV burden is high. nHFBC delivery models are recent strategies and at present resource constraints make this a challenge in many sub-Saharan African settings. The heterogeneity of these nHFBC models in our review ranged from the diversity of the models, be definition and the evaluation methods. Of the 21 articles that were included for inclusion only 15 articles compared outcomes with HFBC, making data available for analysis limited, and its inclusion in the meta-analysis imperfect. Instead of comparing outcomes from every individual nHFBC model to HFBC model separately, the results were pooled, and all community-based programs were evaluated against the standard of care causing clinical heterogeneity. Another limitations in this review include the heterogeneity of the articles that met our inclusion criteria which could have manifested in several ways. Our topic was diverse and the methods of evaluating nHFBC outcomes ranged from facility-site, observational cohorts to randomized trials. With regards to studies reporting on mortality in our review, two observational studies did a comparison between patients who chose nHFBC or not [51, 54] and one study did a comparison in two different settings [56] which could have resulted in bias due to the fact that whether participants received nHFBC or HFBC was not allocated at random. The reported effect estimates were adjusted for potential confounders to mitigate this. Although some residual confounding may remain, the effect observed in the observational cohorts is consistent with that seen in the randomized studies. Assessing outcomes such as LTFU in our review was also a limitation. The lack of a standard definition for LTFU across studies included in our review made it difficult to assess the trends and differences in LFTU to accurately measure the effectiveness of these programs and obstructed comparibility between HFBC and nHFBC models. LTFU is an important indicator to accurately measure effectiveness of ART programs and therefore there is need for a standard definition in order to understand the changes within and the differences between ART programs especially in settings where ascertainment of mortality is weak. Lastly, unsuccessful pilot studies are less likely to be published, introducing publication bias. Studies included in this review introduce bias in measured outcomes in that those included with available data may differ in terms of stability, ability to access care and treatment or being able to make a choice. The value of such nHFBC models for people currently not retained in care is not included in this systematic review. Other limitations include the diversity of the set-up of these nHFBC models and the study design, resulting in observation bias, and confounding bias when a comparison was made. In this review, stable patients were offered the chance or were able to choose themselves and both avenues introduce significant selection bias, as both these groups are likely to contain individuals more dedicated to their health, evident from their superior clinical outcomes or willingness to participate actively in their care.

Although our findings have shown that nHFBC models can complement HFBC service delivery with regards to clinical outcomes and enhance patients ability to manage HIV, there is need for more in depth information on patients acceptability towards these models of care as well as the negative and positive effects related to stigma and ART delivery in the communities [35].

All the articles in our review, with exception of one [42], focused on stable adult PLHIV on ART, which typically included being on ART for more than 6–12 months and either virally suppressed or immunologically stable. However, there is a need to understand the impact of nHFBC models on key populations who are frequently excluded, such as youth and men who have sex with men, who may benefit the most as they may avoid clinics for other reasons such as domestic violence. There is no data regarding nHFBC models towards key populations and further pilot studies on nHFBC models should be targeted towards key populations to determine the feasibility and key clinical outcomes. In additon to the models included in this review, there is a growing trend towards supporting ART distribution from drop-in centres, and therefore a need to assess their effectiveness. However, at the time of evalaution, there were no RCTs that included this approach to explore their outcomes. There is currently scarce or no data regarding patient satisfaction and improvement in quality of life from these models and therefore further research is needed to determine patient satisfaction and quality of life from these models. Feasibility of implementing these models equally need to be explored as most of these models are implemented by in-country implementing partners with additional funding and resources, and need to understand how these models can be placed into the context of existing healthcare sytem without external funding.

## Conclusions

This systematic review further demonstrates non-inferiority of nHFBC amongst stable PLWH on ART in high HIV burden, resource limited settings in sub-Saharan Africa for key outcome measures of VS, death or LTFU compared with current standard HFBC models.

## Supplementary Information

**Additional file 1: Appendix 1.** Search strategy in full for all databases, including Medline, Embase and Global Health

**Additional file 2: Appendix 2: Table 2.**Quality Assessment of Randomised Control Trials using Cochrane Tool for Risk of Bias

**Additional file 3: Appendix 3:** **Table 3.** Quality Assessment of Included Cohort Studies using the Newcastle-Ottawa Scale

**Additional file 4: Appendix 4: Figure 4.** Forest plot for estimated pooled risk difference comparing viral suppression among those receiving health facility based care (HFBC) and non-health facility based care (nHFBC), including results from randomized controlled trials and observational studies. Information on file format. Brief description of file content.

**Additional file 5: Appendix 5.** Synthesis Without Meta-analysis (SWiM) reporting items

**Additional file 6: Appendix 6: Table 4.** Loss to Follow-Up and Retention outcomes of nHFBC and HFBC comparison. **Table 5.** Loss to Follow-Up and Retention outcomes without nHFBC and HFBC comparison.

## Data Availability

The datasets used and/or analysed during the current study available from the corresponding author on reasonable request.

## Abbreviations

AC: Adherence clubs
ART: Antiretroviral treatment
CAG: Community adherence group
CBDP: Community-based distribution group
CHW: Community health worker
DMD: Decentrlaized medication delivery
HFBC: Health facility based care
HIV: Human immunodeficiency virus
LTFU: Loss to follow up
MSF: Médecins Sans Frontiéres
nHFBC: Non-facility based care
PLHIV: People living with HIV
RCT: Randomised control trial
SSA: Sub-Saharan Africa
VL: Viral load
VS: Viral suppression
