# Supplementary material for: A systematic review of the effectiveness of non- health facility based care delivery of antiretroviral therapy for people living with HIV in sub-Saharan Africa measured by viral suppression, mortality and retention on ART
Source: BMC Public Health. 2021 Jun 10;21:1110. doi: 10.1186/s12889-021-11053-8 (PMC8194040; doi:10.1186/s12889-021-11053-8)
Supplement: Supplementary file 1 — Additional file 1: Appendix 1. Search strategy in full for all databases, including Medline, Embase and Global Health [file 12889_2021_11053_MOESM1_ESM.docx]

**Appendix 1:**

**Database: Medline**

**Search Strategy:**

-------------------------------------------------------------------------------------------------------------

| 1. Antiretroviral Therapy, Highly Active/ |  |
| --- | --- |
| 2. exp Anti-Retroviral Agents/ |  |
| 3. (Antiretroviral therap* or Anti retroviral therap* or Antiretroviral treat* or Anti retroviral treat* or HAART or ART or Anti Retroviral Agent* or Antiretroviral Agent* or antiretroviral deliver* or anti retroviral deliver*).ab,ti. |  |
| 4. 1 or 2 or 3 |  |
| 5. exp "Africa South of the Sahara"/ |  |
| 6. (Sub-Saharan Africa or Subsaharan Africa).ab,ti. |  |
| 7. (Angola or Gambia or Nigeria or Benin or Dahomey or Ghana or Gold Coast or Rwanda or Ruanda or Botswana or Bechuanaland or Kalahari or Guinea or St Helena or Saint Helena or Burkina Fas?o or Upper Volta or Senegal or Burundi or Urundi or Kenya or Seychelles or Cameroon* or Lesotho or Basutoland or Sierra Leone or Cape Verde or Liberia or Somalia or Central African Republic or Ubangi-Shari or Madagascar or Malagasy or South Africa or Chad or Malawi or Nyasaland or Sudan or Comoro* or Iles Comores or Mayotte or Mali or Swaziland or Congo or Kinshasa or Zaire or Katanga or Mauritania or Tanzania or Zanzibar or Tanganyika or Brazzaville or Mauritius or Agalega or Togo or Togolese or Cote dIvoire or Cote d Ivoire or Ivory Coast or Uganda or Eritrea or Mozambique or Portuguese East Africa or Zambia or Rhodesia or Ethiopia or Namibia or Zimbabwe or Gabon or Gabonese Republic or Niger).ab,ti. |  |
| 8. (Sao Tome and Principe).ab,ti. |  |
| 9. 5 or 6 or 7 or 8 |  |
| 10. Home Health Aides/ |  |
| 11. Volunteers/ |  |
| 12. Home Nursing/ |  |
| 13. Peer Group/ |  |
| 14. Social Support/ |  |
| 15. Social Welfare/ |  |
| 16. Community Integration/ |  |
| 17. Community Health Workers/ |  |
| 18. Home Care Services/ |  |
| 19. Community Pharmacy Services/ |  |
| 20. (Community adj3 (Health service* or Health Care or Healthcare or ART or antiretroviral therap* or anti retroviral therap*)).ab,ti. |  |
| 21. (Home base* or Homebase* or Community based).ab,ti. |  |
| 22. ((Community or Adherence or Peer) adj4 (Program* or support or group* or club* or service* or treatment*)).ab,ti. |  |
| 23. ((community or village? or peer) adj3 (health worker? or health care worker? or healthcare worker? or Healthworker? or Health Personnel or Health Care Provider* or Healthcare Provider* or service* or refill or Decentrali#ation or Shift*)).ab,ti. |  |
| 24. (community adj3 (volunteer? or aide or aides)).ab,ti. |  |
| 25. (Community adj8 distribution point*).ab,ti. |  |
| 26. (Treatment partner* or Fieldworker* or Field Worker* or scale up or scaling up).ab,ti. |  |
| 27. 10 or 11 or 12 or 13 or 14 or 15 or 16 or 17 or 18 or 19 or 20 or 21 or 22 or 23 or 24 or 25 or 26 |  |
| 28. 4 and 9 and 27 |  |
| 29. limit 28 to yr="2010 -Current" |  |

**Database: Embase**

**Search Strategy:**

---------------------------------------------------------------------------------------------------------------------

| 1. exp highly active antiretroviral therapy/ |  |
| --- | --- |
| 2. exp antiretrovirus agent/ |  |
| 3. (Antiretroviral therap* or Anti retroviral therap* or Antiretroviral treat* or Anti retroviral treat* or HAART or ART or Anti Retroviral Agent* or Antiretroviral Agent* or antiretroviral deliver* or anti retroviral deliver*).ab,ti. |  |
| 4. 1 or 2 or 3 |  |
| 5. exp "africa south of the sahara"/ or angola/ or benin/ or botswana/ or burkina faso/ or burundi/ or cameroon/ or cape verde/ or central africa/ or central african republic/ or chad/ or comoros/ or congo/ or cote d'ivoire/ or democratic republic congo/ or djibouti/ or equatorial guinea/ or eritrea/ or ethiopia/ or gabon/ or gambia/ or ghana/ or guinea/ or guinea-bissau/ or kenya/ or lesotho/ or liberia/ or madagascar/ or malawi/ or mali/ or mayotte/ or mozambique/ or namibia/ or niger/ or nigeria/ or rwanda/ or senegal/ or sierra leone/ or somalia/ or south africa/ or sudan/ or swaziland/ or tanzania/ or togo/ or uganda/ or zambia/ or zimbabwe/ |  |
| 6. (Sub-Saharan Africa or Subsaharan Africa).ab,ti. |  |
| 7. (Angola or Gambia or Nigeria or Benin or Dahomey or Ghana or Gold Coast or Rwanda or Ruanda or Botswana or Bechuanaland or Kalahari or Guinea or St Helena or Saint Helena or Burkina Fas?o or Upper Volta or Senegal or Burundi or Urundi or Kenya or Seychelles or Cameroon* or Lesotho or Basutoland or Sierra Leone or Cape Verde or Liberia or Somalia or Central African Republic or Ubangi-Shari or Madagascar or Malagasy or South Africa or Chad or Malawi or Nyasaland or Sudan or Comoro* or Iles Comores or Mayotte or Mali or Swaziland or Congo or Kinshasa or Zaire or Katanga or Mauritania or Tanzania or Zanzibar or Tanganyika or Brazzaville or Mauritius or Agalega or Togo or Togolese or Cote dIvoire or Cote d Ivoire or Ivory Coast or Uganda or Eritrea or Mozambique or Portuguese East Africa or Zambia or Rhodesia or Ethiopia or Namibia or Zimbabwe or Gabon or Gabonese Republic or Niger).ab,ti. |  |
| 8. (Sao Tome and Principe).ab,ti. |  |
| 9. 5 or 6 or 7 or 8 |  |
| 10. exp voluntary worker/ |  |
| 11. exp home care/ |  |
| 12. exp peer group/ |  |
| 13. exp social support/ |  |
| 14. exp social welfare/ |  |
| 15. exp community integration/ |  |
| 16. exp health auxiliary/ |  |
| 17. (Community adj3 (Health service* or Health Care or Healthcare or ART or antiretroviral therap* or anti retroviral therap*)).ab,ti. |  |
| 18. (Home base* or Homebase* or Community based).ab,ti. |  |
| 19. ((Community or Adherence or Peer) adj4 (Program* or support or group* or club* or service* or treatment*)).ab,ti. |  |
| 20. ((community or village? or peer) adj3 (health worker? or health care worker? or healthcare worker? or Healthworker? or Health Personnel or Health Care Provider* or Healthcare Provider* or service* or refill or Decentrali#ation or Shift*)).ab,ti. |  |
| 21. (community adj3 (volunteer? or aide or aides)).ab,ti. |  |
| 22. (Community adj8 distribution point*).ab,ti. |  |
| 23. home health aides.ab,ti. |  |
| 24. community pharmacy services.ab,ti. |  |
| 25. (Treatment partner* or Fieldworker* or Field Worker* or scale up or scaling up).ab,ti. |  |
| 26. 10 or 11 or 12 or 13 or 14 or 15 or 16 or 17 or 18 or 19 or 20 or 21 or 22 or 23 or 24 or 25 |  |
| 27. 4 and 9 and 26 |  |
| 28. limit 27 to yr="2010 -Current" |  |

**Database: Global Health**

**Search strategy**

| 1. exp highly active antiretroviral therapy/ |  |
| --- | --- |
| 2. exp antiretroviral agents/ |  |
| 3. (Antiretroviral therap* or Anti retroviral therap* or Antiretroviral treat* or Anti retroviral treat* or HAART or ART or Anti Retroviral Agent* or Antiretroviral Agent* or antiretroviral deliver* or anti retroviral deliver*).ab,ti. |  |
| 4. 1 or 2 or 3 |  |
| 5. exp "Africa South of Sahara"/ |  |
| 6. (Sub-Saharan Africa or Subsaharan Africa).ab,ti. |  |
| 7. (Angola or Gambia or Nigeria or Benin or Dahomey or Ghana or Gold Coast or Rwanda or Ruanda or Botswana or Bechuanaland or Kalahari or Guinea or St Helena or Saint Helena or Burkina Fas?o or Upper Volta or Senegal or Burundi or Urundi or Kenya or Seychelles or Cameroon* or Lesotho or Basutoland or Sierra Leone or Cape Verde or Liberia or Somalia or Central African Republic or Ubangi-Shari or Madagascar or Malagasy or South Africa or Chad or Malawi or Nyasaland or Sudan or Comoro* or Iles Comores or Mayotte or Mali or Swaziland or Congo or Kinshasa or Zaire or Katanga or Mauritania or Tanzania or Zanzibar or Tanganyika or Brazzaville or Mauritius or Agalega or Togo or Togolese or Cote dIvoire or Cote d Ivoire or Ivory Coast or Uganda or Eritrea or Mozambique or Portuguese East Africa or Zambia or Rhodesia or Ethiopia or Namibia or Zimbabwe or Gabon or Gabonese Republic or Niger).ab,ti. |  |
| 8. (Sao Tome and Principe).ab,ti. |  |
| 9. 5 or 6 or 7 or 8 |  |
| 10. exp home health aides/ |  |
| 11. exp community health services/ |  |
| 12. exp medical auxiliaries/ |  |
| 13. exp home care/ |  |
| 14. exp peer influence/ |  |
| 15. exp peer relationships/ |  |
| 16. exp support systems/ |  |
| 17. exp social welfare/ |  |
| 18. exp social integration/ |  |
| 19. exp community health services/ |  |
| 20. (Community adj3 (Health service* or Health Care or Healthcare or ART or antiretroviral therap* or anti retroviral therap*)).ab,ti. |  |
| 21. (Home base* or Homebase* or Community based).ab,ti. |  |
| 22. ((Community or Adherence or Peer) adj4 (Program* or support or group* or club* or service* or treatment*)).ab,ti. |  |
| 23. ((community or village? or peer) adj3 (health worker? or health care worker? or healthcare worker? or Healthworker? or Health Personnel or Health Care Provider* or Healthcare Provider* or service* or refill or Decentrali#ation or Shift*)).ab,ti. |  |
| 24. (community adj3 (volunteer? or aide or aides)).ab,ti. |  |
| 25. (Community adj8 distribution point*).ab,ti. |  |
| 26. (Treatment partner* or Fieldworker* or Field Worker* or scale up or scaling up).ab,ti. |  |
| 27. 10 or 11 or 12 or 13 or 14 or 15 or 16 or 17 or 18 or 19 or 20 or 21 or 22 or 23 or 24 or 25 or 26 |  |
| 28. 4 and 9 and 27 |  |
| 29. limit 28 to yr="2010 -Current" |  |
