# Supplementary material for: A systematic review of the effectiveness of non- health facility based care delivery of antiretroviral therapy for people living with HIV in sub-Saharan Africa measured by viral suppression, mortality and retention on ART
Source: BMC Public Health. 2021 Jun 10;21:1110. doi: 10.1186/s12889-021-11053-8 (PMC8194040; doi:10.1186/s12889-021-11053-8)
Supplement: Supplementary file 2 — Additional file 2: Appendix 2: Table 2.Quality Assessment of Randomised Control Trials using Cochrane Tool for Risk of Bias [file 12889_2021_11053_MOESM2_ESM.docx]

**Appendix 2**

| **Table 2: Quality Assessment of Randomised Control Trials using Cochrane Tool for Risk of Bias** | | | | | | |
| --- | --- | --- | --- | --- | --- | --- |
|  | **Fox** | **Selke** | **Hanrahan** | **Geldsetzer** | **Woodd** | **Amuron** |
| **Sequence Generation** |  |  |  |  |  |  |
| **Allocation concealment** |  |  |  |  |  |  |
| **Blinding (participants & personnel)** |  |  |  |  |  |  |
| **Blinding (outcome assessment)** |  |  |  |  |  |  |
| **Incomplete outcome data** |  |  |  |  |  |  |
| **Selective reporting** |  |  |  |  |  |  |
| **Other bias** |  |  |  |  |  |  |

***Table 2 Legend***

 = low risk of bias = high risk of bias = not enough information to determine
