# Supplementary material for: A systematic review of the effectiveness of non- health facility based care delivery of antiretroviral therapy for people living with HIV in sub-Saharan Africa measured by viral suppression, mortality and retention on ART
Source: BMC Public Health. 2021 Jun 10;21:1110. doi: 10.1186/s12889-021-11053-8 (PMC8194040; doi:10.1186/s12889-021-11053-8)
Supplement: Supplementary file 3 — Additional file 3: Appendix 3: Table 3. Quality Assessment of Included Cohort Studies using the Newcastle-Ottawa Scale [file 12889_2021_11053_MOESM3_ESM.docx]

**Appendix 3**

| **Table 3: Quality Assessment of Included Cohort Studies using the Newcastle-Ottawa Scale** | | | | | | | | | |
| --- | --- | --- | --- | --- | --- | --- | --- | --- | --- |
| **Study** | **Selection*** | | | | **COMPARABILITY**** | **Outcome***** | | | **Total Stars** |
|  | Representat-iveness of exposed cohort | Selection of non-exposed cohort | Ascertainment of exposure | Demonstration that outcome not present at the start |  | Assessment of outcome | Long follow-up | Adequacy of follow-up |  |
| Fox 2019^(37)^ | ★ | ★ | ★ |  | ★ | ★ | ★ | ★ | **7** |
| Tun 2019^(48)^ | ★ | ★ |  | ★ | ★ |  |  | ★ | **5** |
| Pasipamire 2018^(49)^ | ★ | ★ | ★ | ★ | ★★ | ★ | ★ | ★ | **9** |
| Myer 2017^(50)^ | ★ | ★ | ★ |  | ★★ | ★ |  | ★ | **7** |
| Vogt 2017^(51)^ § | - | - | - | - | - | - | - | - | **-** |
| Tsondai 2017^(52)^ § | - | - | - | - | - | - | - | - | **-** |
| Decroo 2017^(56)^ | ★ | ★ | ★ | ★ | ★ | ★ | ★ | ★ | **8** |
| Auld 2016^(53)^ | ★ | ★ | ★ | ★ | ★ | ★ | ★ |  | **7** |
| Grimsrud 2016^(54)^ | ★ | ★ | ★ |  | ★ | ★ |  | ★ | **6** |
| Okoboi 2016^(55)^ | ★ | ★ | ★ | ★ | ★★ | ★ | ★ |  | **8** |
| Jobarteh 2016^(57)^ | ★ | ★ | ★ | ★ | ★ | ★ |  | ★ | **7** |
| Okoboi 2015^(36)^ § | - | - | - | - | - | - | - | - | **-** |
| Decroo 2014^(32)^ § | - | - | - | - | - | - | - | - | **-** |
| Luque-Fernandez^(58)^ 2013 | ★ | ★ | ★ |  | ★ | ★ | ★ | ★ | **7** |
| Kipp 2012^(59)^ | ★ | ★ | ★ |  | ★ | ★ | ★ | ★ | **7** |

**
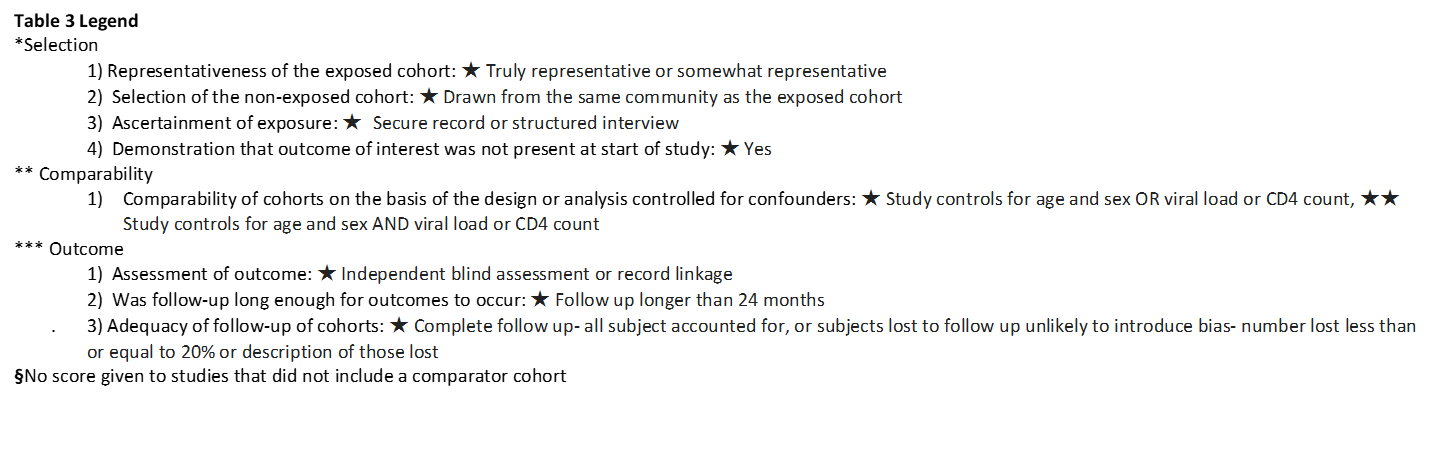
**

**Quality Assessment**

Quality assessment of the 6 randomised control trials was done using the Cochrane Risk of Bias Tool(41). The risk of bias in the eight different categories was generally determined to be low for most studies. Selection bias was low for most studies rated through the sequence generation and allocation concealment. Reportive bias was determined to be low, and many studies had a study protocol published. Detection bias was reduced as many of the outcome assessors were blinded through follow up of patient through anonymized records. Attrition bias was determined to be low for all studies due to relatively low loss to follow up rates. The exception was high risk of performance bias, as none of the studies were able to blind the participants and personnel, due to the nature of the intervention. Other sources of bias were difficult to determine. Quality assessment of the 15 cohort studies was done using the Newcastle-Ottawa Scale (41). Four out of the 15 cohort studies (32, 36, 51, 52) did not have a control group and were therefore not evaluated using the scale. Of the cohort studies that did have a comparator group, 10 of the cohort studies were rated as Good Quality, 1 was rated as Fair Quality and none were rated as Poor Quality as per the AHRQ standards. Being stable on ART was an inclusion criterion for most community interventions, which was acknowledged by the papers to be a confounding factor. Therefore, these cohorts were considered to be somewhat representative. Comparability of cohorts were generally adequate with similar baseline characteristics. Quality was maintained by using records rather than self-reporting in the ascertainment of exposure to the intervention and assessment of outcomes in all but one cohort study. Furthermore, loss of follow up rates were below 20% for the majority of studies. The follow up duration for outcomes was more than 24 months for 10 of the 16 articles.
