# Supplementary material for: A systematic review of the effectiveness of non- health facility based care delivery of antiretroviral therapy for people living with HIV in sub-Saharan Africa measured by viral suppression, mortality and retention on ART
Source: BMC Public Health. 2021 Jun 10;21:1110. doi: 10.1186/s12889-021-11053-8 (PMC8194040; doi:10.1186/s12889-021-11053-8)
Supplement: Supplementary file 4 — Additional file 4: Appendix 4: Figure 4. Forest plot for estimated pooled risk difference comparing viral suppression among those receiving health facility based care (HFBC) and non-health facility based care (nHFBC), including results from randomized controlled trials and observational studies. Information on file format. Brief description of file content. [file 12889_2021_11053_MOESM4_ESM.docx]

**Appendix 4**

***Fig 4. Forest plot for estimated pooled risk difference comparing viral suppression among those receiving health facility based care (HFBC) and non-health facility based care (nHFBC), including results from randomized controlled trials and observational studies.***


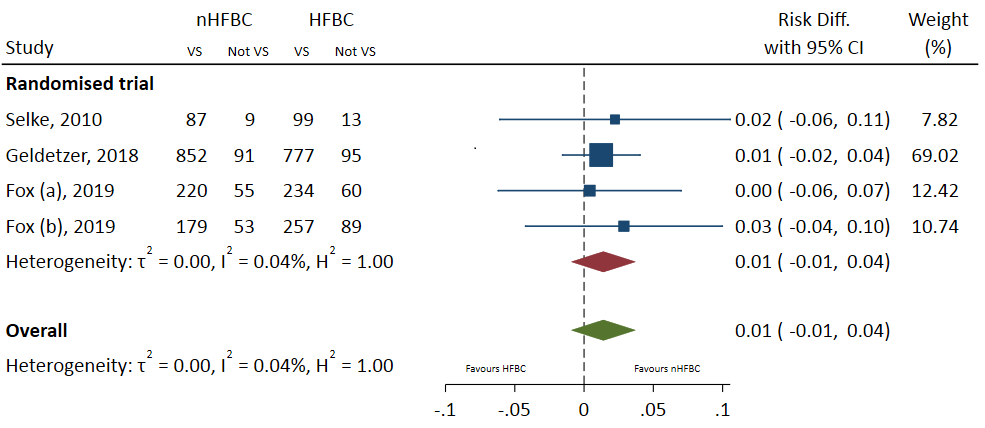


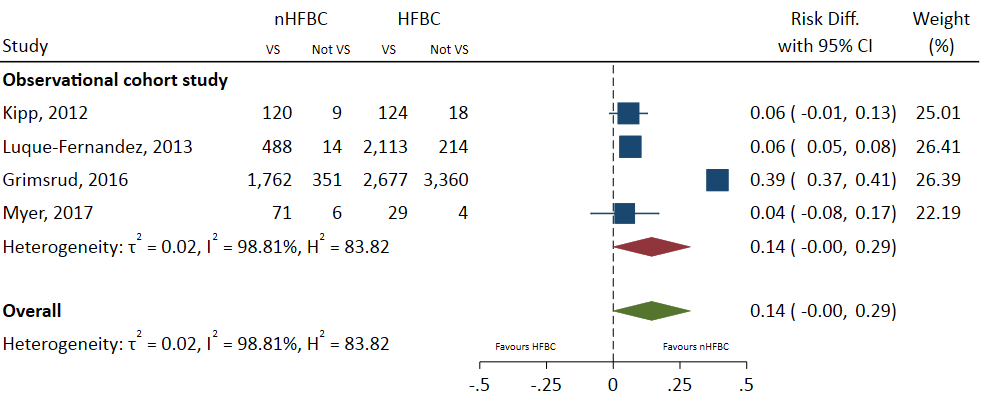


***VS – virally suppressed***
