# Supplementary material for: A systematic review of the effectiveness of non- health facility based care delivery of antiretroviral therapy for people living with HIV in sub-Saharan Africa measured by viral suppression, mortality and retention on ART
Source: BMC Public Health. 2021 Jun 10;21:1110. doi: 10.1186/s12889-021-11053-8 (PMC8194040; doi:10.1186/s12889-021-11053-8)
Supplement: Supplementary file 6 — Additional file 6: Appendix 6: Table 4. Loss to Follow-Up and Retention outcomes of nHFBC and HFBC comparison. Table 5. Loss to Follow-Up and Retention outcomes without nHFBC and HFBC comparison. [file 12889_2021_11053_MOESM6_ESM.docx]

**Appendix 6**

**Table 4: Loss to Follow-Up and Retention outcomes of nHFBC compared to HFBC**

| Source | Country | Model Name | Follow-up time | Outcome definition | Findings |
| --- | --- | --- | --- | --- | --- |
| LOSS TO FOLLOW-UP | | | | | |
| Hanrahan  2019^(44)^ | South Africa | Community Adherence Clubs | 24 months | Patients who missed a club visit and did not pick up ART medications within 5 days, had 2 consecutive late ART pick-ups, developed a comorbidity or had viral rebound were referred to standard of care | During the 24 months follow up, there was no significant difference in loss to ART between clinic and community clubs.  12% among community club’s vs 7% among facility clubs [HR 1.69, 95%CI 0.98, 2.91]  Overall, 10% loss from ART clubs |
| Geldsetzer  2018^(43)^ | Tanzania | Home Delivery model | 326 days | Patients in the intervention arm who did not have a VL measurement after enrolment were considered LTFU | 18.9% in Home delivery model vs 13.6% in HCF |
| Selke  2010^(46)^ | Kenya | Home Delivery model | 28 months | Point at which the person was no longer in care (moved out, quit medications or shifted to another facility). | LTFU was 5.2% in Home delivery model and 4.5% in HCF. |
| Wood  2014^(45)^ | Uganda | Home Delivery Model | 28 months | Participants who were no longer in care during the study period | 1.8% among those with CD4 <50 and 2.6% among those with CD4 > 50cells |
| Grimsrud  2016^(54)^ | South Africa | Community Adherence clubs | 12 months | LTFU defined as having no visit in the first 12 weeks (excluding mortality) after analysis closure.  Analysis closure was at the end of 2013 and database closure was 24^th^ March 2014. LTFU was defined as having no visit in the in the first 12 weeks of 2014. | Community clubs were associated with a substantial decrease in the risk of LTFU compared with the community clinic.  However, LTFU was twice as likely in youths compared to older patients.  Clubs in the community (CAC) were associated with a reduction in the risk of LTFU compared with clinic with a two-third reduction in the hazard of LTFU |
| Auld  2016^(53)^ | Mozambique | Community ART Support Group (CASG) | 4 years | LTFU was defined as > 60 days late for their next scheduled drug pick up. | Participating in CASG was associated with a 35% lower LTFU rates (AHR 0.65; 95%CI: 0.46, 0.91).  LTFU incidence was 2.9% at 2 yrs. and 10.1% at 4 years.  In a sensitivity analysis, when restricting the cohorts to the clinics that only offered CASG models during the 4 years of follow-up, CASG participation was associated with a 55% reduction in LTFU rates [AHR 0.45 95%CI 0.32-0.64] |
| Decroo  2017^(56)^ | Mozambique | Community Adherence groups (CAGs) | 4 years | LTFU was defined as being more than 2 months overdue for their most recent appointment or scheduled ART refill. | Combined LTFU and mortality  CAG members had a greater than fivefold reduction in the risk of dying or being LTFU [ AHr 0.18; 95%CI 0.11, 0.29] |
| Jobarteh  2016^(57)^ | Mozambique | Community ART Support groups (CASG) | 12 months | LTFU > 60 days late for their next scheduled appointment | LTFU among CASG and non-CASG members were 7.25 and 15.9% respectively.  Non-CASG members had significantly higher LTFU [HR 2.36 95%CI: 1.54, 3.17] |
| Luque Fernandez  2013^(58)^ | South Africa | Community Adherence Clubs | 3 years | Combined outcome of time to either death or LTFU.  LTFU – not having any contact with the service in the 6 months following analysis closure | 12.8% were LTFU or died.  Both outcomes less frequent in club members [Crude RR 0.25 95%CI: 0.14-0.41] |
| Tun 2019^(48)^ | Tanzania | Community distribution points (CDP) | 6 months | LTFU was defined as any patients who had died, transferred out or withdrew from the model | 53 in the intervention arm and 55 in the HCF |
|  |  |  |  |  |  |
|  | | | | | |
| RETENTION | | | | | |
| Fox 2019^(37)^ | South Africa | Adherence clubs | 12 months | Retention in care at 12 months after model eligibility. Defined as 100% - % attrition, with attrition as the sum of reported deaths, LTFU and transfers. LTFU was defined as failure to attend the clinic within 90 days of a scheduled appointment. | ACs had a higher retention rate  81.6% participants retained in facility; 89.5% participants retained in the community. Risk difference 7.8% (95% CI; 2.1%, 13.6%). |
| Fox 2019^(37)^ | South Africa | Community distribution points (CDP) | 12 months | Retention in care at 12 months after model eligibility. Defined as 100% - % attrition, with attrition as the sum of reported deaths, LTFU and transfers. LTFU was defined as failure to attend the clinic within 90 days of a scheduled appointment. | Retention was high overall (about 85%)  87.2% patients retained in the facility; 81.5% patients retained in the community. Risk difference -5.8% (95% CI; -11.75%, 0.2%). |
| Jobarteh 2016^(57)^ | Mozambique | Community ART support groups (CASG) | 12 months | Retention defined as patients who were in care at the end of 12 months [excludes LTFU] | Overall patients in CASG had better retention rates  Having excluded those who were LTFU and died the number of patients retained in CASG was 91.4% and 82.9% in Non-CASG models |
| Decroo  2017^(56)^ | Mozambique | Community ART support groups (CASG) | 24 months | Patients retained in care [excluding those LTFU or died] | Retention in care among patients in CAGs was substantially higher than those in individual care.  Overall RIC was 90.8% at 12 months and 86% at 24 months.  At 12 months: 89.5 % retained in care (95% CI; 87.9, 90.8) in the facility, 99.1% retained in care (95% CI; 97.3, 99.7) in the community.  At 24 months 82.3 % retained in care (95% CI: 79.9, 84.5) in the facility, and 97.5% retained in care (95% CI; 95.4, 98.6) in the community. |
| Kipp 2012^(59)^ | Uganda | Home delivery | 24 months | Patients who remained active in care at the end of the study period [ excludes LTFU and mortality] | 71% were retained in care in the health care facility and 70% retained in the home delivery model |
|  |  |  |  |  |  |
|  |  |  |  |  |  |
| Source | **Country** | **Model name** | **Follow-up time** | **Outcome definition** | **Findings** |
| Tun 2019^(48)^ | Tanzania | Community distribution points (CDP) | 6 months | Patients active in care at 6 months | 82.8% patients retained in CDP models at 6 months vs 82.1% retained in the facility. No formal analysis done. |
| Okoboi 2016^(55)^ | Uganda | Community distribution points (CDP) | 5 years | Retention was defined as any patient who had at least one clinic visit in the six months before June 2013; was still alive at the end of June 2013, excluding those deaths reported to TASO stopped ART; or LTFU. | 83.9% retained in the facility, 82.9% retained in the community. P value 0.670. Univariate analysis of factors associated with attrition: 1.00 (0.76-1.34), P value 0.972. |
| Selke 2010^(46)^ | Kenya | Home ART delivery | 28 months | Defined as point at which patient was no longer in care (transfer, quit medications] | 91.1% retained in the facility and 90.6% retained in the community – no formal analysis |
| Geldsetzer^*^ 2018^(43)^ | Tanzania | Home ART delivery | 326 days | Patients who were still active in care | 81.1% retained in the community and 86.4% retained in the facility – no formal analysis done. |

**Legend:** ** The inverse numbers of attrition reported here as retention*

**Table 5: Loss to Follow-Up and Retention outcomes of nHFBC without HFBC comparison**

| Source | Country | Model Name | Follow-up time | Outcome definition | Findings |
| --- | --- | --- | --- | --- | --- |
|  |  |  |  |  |  |
| LOSS TO FOLLOW-UP | | | | | |
| Vogt 2017^(51)^ | Democratic Republic of Congo | Community based refill centre | 24 months | Defined death and LTFU as attrition  LTFU was defined as having had no contact with the services between 2011 and 2013 | LTFU was 9.0% at 24 months  Deaths were not well captured so could have overestimated the LTFU rates and reduced mortality rates. |
| Tsondai  2017^(52)^ | South Africa | Adherence clubs | 24 months | LTFU was defined as having no contact with the club or clinic in the 6 months following analysis closure and was determined to have happened on the date of last contact with service | 4.2% of patients were LTFU  Cumulative incidence of LTFU was:  2.6% [95%CI 2.1-3.2] at 12 months  12.2% [95%CI 9.7, 14.7] at 36 months  Risk of LTFU was observed in younger patients |
| Okoboi 2015^(36)^ | Uganda | Community distribution points (CDP) | 5 years | Combined mortality and LTFU  LTFU was defined as having had no visit or contact with the service during the study period | LTFU 1.59 per 100-person per years |
|  |  |  |  |  |  |
| Decroo 2014^(32)^ | Mozambique | Community Adherence Groups (CAGs) | 4 years | LTFU defined as being more than 2 months late for the last appointment/ refill | LTFU rate was 0.1 per 100 person yrs. |
| Pasipamire 2018^(49)^ | Swaziland | Community adherence groups (CAGs)  Facility based groups  Treatment outreach | 12 months | Patient LTFU was defined as patients without recorded visit for 120 days or more before Database closure. LTFU from care was time from enrolment to the composite endpoint of LTFU and death, regardless whether the outcome occurred while enrolled in the care model or in routine facility-based ART care | Of the 918 total patients included, 27 were LTFU (2.94%). Patients in CAGs had a higher risk of disengaging from care models (aHR 3.15 95%CI 2.01, 4.95)  Note this was comparison between two models and not the facility |
|  |  |  |  |  |  |
| RETENTION | | | | | |
| Pasipamire  2018^(49)^ | Swaziland | Community adherence groups (CAGs)  Facility based groups  Treatment outreach | 12 months | In the primary analysis, Retention in care model, the outcome of interest was time to the composite endpoint of LTFU, death or exit from specific care model at enrolment.  In the secondary analysis, Retention in ART care, outcome was time from enrolment to the composite endpoint of LTFU and death regardless of whether the outcome occurred whilst enrolled in the model of care or routine HCF. | **Retention in the care models**  Overall care model retention was 90.9% at 6 months and 82.2% at 12 months. Retention in care models differed significantly by model types, lowest in CAGs. 70.4% retained in CAGs at 12 months compared to 86.3% in outreach and 90.4% in clubs. Patients in CAGs had a higher risk of disengaging from care models compared with treatment clubs [adjusted HR 3.15, 95% ci 2.01, 4.95].  **Retention in ART**  Overall, ART retention was 96.7% at 6 months and 93.7% at 12 months. It was over 90% for all 3 models at all time points and no difference between care models |
| Tsondai 2017^(52)^ | South Africa | Adherence clubs | 12 months  24 months | Primary outcome was LTFU and viral rebound  Competing risk regression was used to estimate the cumulative incidence for LTFU, transfer out and mortality which were then used to calculate the corresponding cumulative retention | Of the 3216 adults contributing 4019 person yrs. follow up, retention was 95.2% at 12 months [ 95% CI 94.0, 96.4] and 89.3% at 24 months [95%CI 87.1, 91.4] after club enrolment. |
| Okoboi  2015^(36)^ | Uganda | Community ART distribution | 5 years | Retention to care was defined as any patient with at least one visit in the 6 months | More than 69% of patients who initiated ART from 2004 to 2009 were retained in care after more than 5 years of treatment. These finding demonstrated that high retention rates are possible even in rural resource limited settings. |
| Decroo 2014^(32)^ | Mozambique | Community Adherence groups (CAGs) | 4 years | Patients who were still active in the models of care at follow up intervals | Retention among CAG members:  At 1 year – 97.7% [95% CI 97.4, 98.2]  At 2 years- 96.0% [95%CI 95.3, 96.6]  At 3 years- 93.4% [95%CI 92.3, 94.3]  AT 4 years- 91.8% [95%CI 90.1, 93.2] |
